# Expanded male sex-determining region conserved during the evolution of homothallism in the green alga *Volvox*

**DOI:** 10.1016/j.isci.2023.106893

**Published:** 2023-06-01

**Authors:** Kayoko Yamamoto, Ryo Matsuzaki, Wuttipong Mahakham, Wirawan Heman, Hiroyuki Sekimoto, Masanobu Kawachi, Yohei Minakuchi, Atsushi Toyoda, Hisayoshi Nozaki

**Affiliations:** 1 Department of Chemical and Biological Sciences, Faculty of Science, Japan Women’s University, Tokyo 112-8681 Japan; 2 Biodiversity Division, National Institute for Environmental Studies, Tsukuba 305-8506, Japan; 3 Department of Biology & Applied Taxonomic Research Center, Faculty of Science, Khon Kaen University, Khon Kaen, Thailand; 4 Department of Science and Mathematics, Faculty of Science and Health Technology, Kalasin University, Mueang Kalasin, Thailand; 5 Department of Genomics and Evolutionary Biology, National Institute of Genetics, Mishima 411-8540, Japan; 6 Department of Biological Sciences, Graduate School of Science, The University of Tokyo, Tokyo 113-0033, Japan

**Keywords:** Chromosome organization, Phylogenetics, Plant evolution

## Abstract

Male and female genotypes in heterothallic (self-incompatible) species of haploid organisms, such as algae and bryophytes, are generally determined by male and female sex-determining regions (SDRs) in the sex chromosomes. To resolve the molecular genetic basis for the evolution of homothallic (bisexual and self-compatible) species from a heterothallic ancestor, we compared whole-genome data from Thai and Japanese genotypes within the homothallic green alga *Volvox africanus*. The Thai and Japanese algae harbored expanded ancestral male and female SDRs of ∼1 Mbp each, representing a direct heterothallic ancestor. Therefore, the expanded male and female ancestral SDRs may originate from the ancient (∼75 mya) heterothallic ancestor, and either might have been conserved during the evolution of each homothallic genotype. An expanded SDR-like region seems essential for homothallic sexual reproduction in *V. africanus*, irrespective of male or female origin. Our study stimulates future studies to elucidate the biological significance of such expanded genomic regions.

## Highlights

• Providing a genome assembly for one of the three bisexual types in *Volvox africanus*
• The assembly harbored expanded, ancestral sex-determining region (SDR) of male origin
• Expanded male or female SDR may be conserved during the evolution of bisexual types
• Ancestral SDR seems essential for bisexuality, regardless of male or female origin

## Graphical abstract

**Figure:**
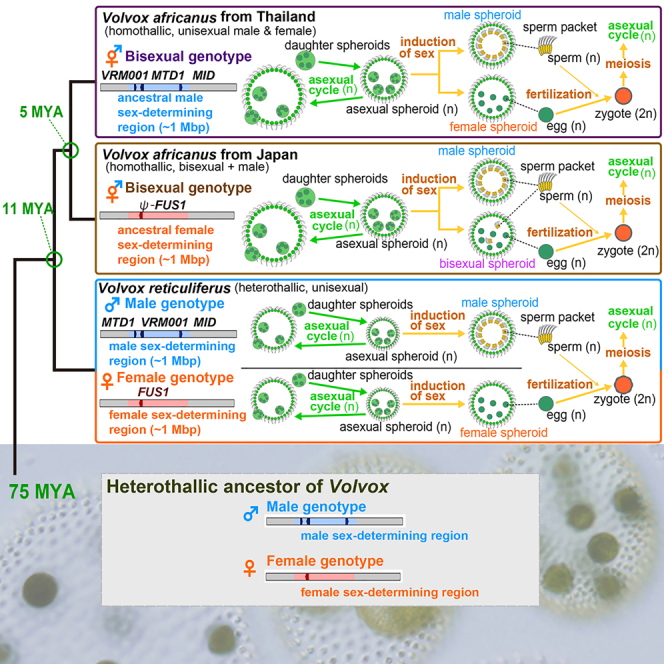

## Introduction

Evolutionary transitions between self-incompatible and self-fertile mating systems have been an exciting topic of evolutionary biology since Charles Darwin.^1^ Extensive studies of the transitions between species with separate sexes and hermaphrodite species in which individuals have both sex functions have been conducted in diploid organisms, such as invertebrates and seed plants.^1^^,^^2^ Recently, how such transitions occurred in the sex-determining regions (SDRs) found in haploid sex chromosomes^3^ of algae and bryophytes was demonstrated by studying whole genomes of Japanese culture strains of two closely related species of the green algal genus *Volvox*^4^: heterothallic (self-incompatible with genetically different male and female) *Volvox reticuliferus* and homothallic (bisexual, with the ability to fertilize within a clone) *V. africanus*. The genome data demonstrated that heterothallic *V. reticuliferus* has an expanded (∼1 Mbp) SDR as found in another heterothallic species *Volvox carteri*, which suggests an ancient (∼75 mya) origin of the expanded SDR within the genus *Volvox*.^4^ Furthermore, homothallic *V. africanus* originating from Japan (*V. africanus* JP) harbors an expanded (∼1 Mbp) sex-determining-like region (SDLR) originating from a female SDR of the ancestral heterothallic species.^4^ However, the culture strain of *V. africanus* JP represents only one of the three mating systems of homothallic *V. africanus*^4^^,^^5^ (homothallic, male-bisexual type; Table S1). Thus, further information on the SDLR (if present) in the other two mating systems of *V. africanus* (Table S1) would be very valuable for understanding the origin of homothallism and the biological importance of the female-derived expanded SDLR in homothallic *V. africanus*.^4^ However, extended genome studies using these two homothallic mating systems seemed impossible because of the unavailability of *V. africanus* culture strains with these two systems.^6^

**Figure 1:**
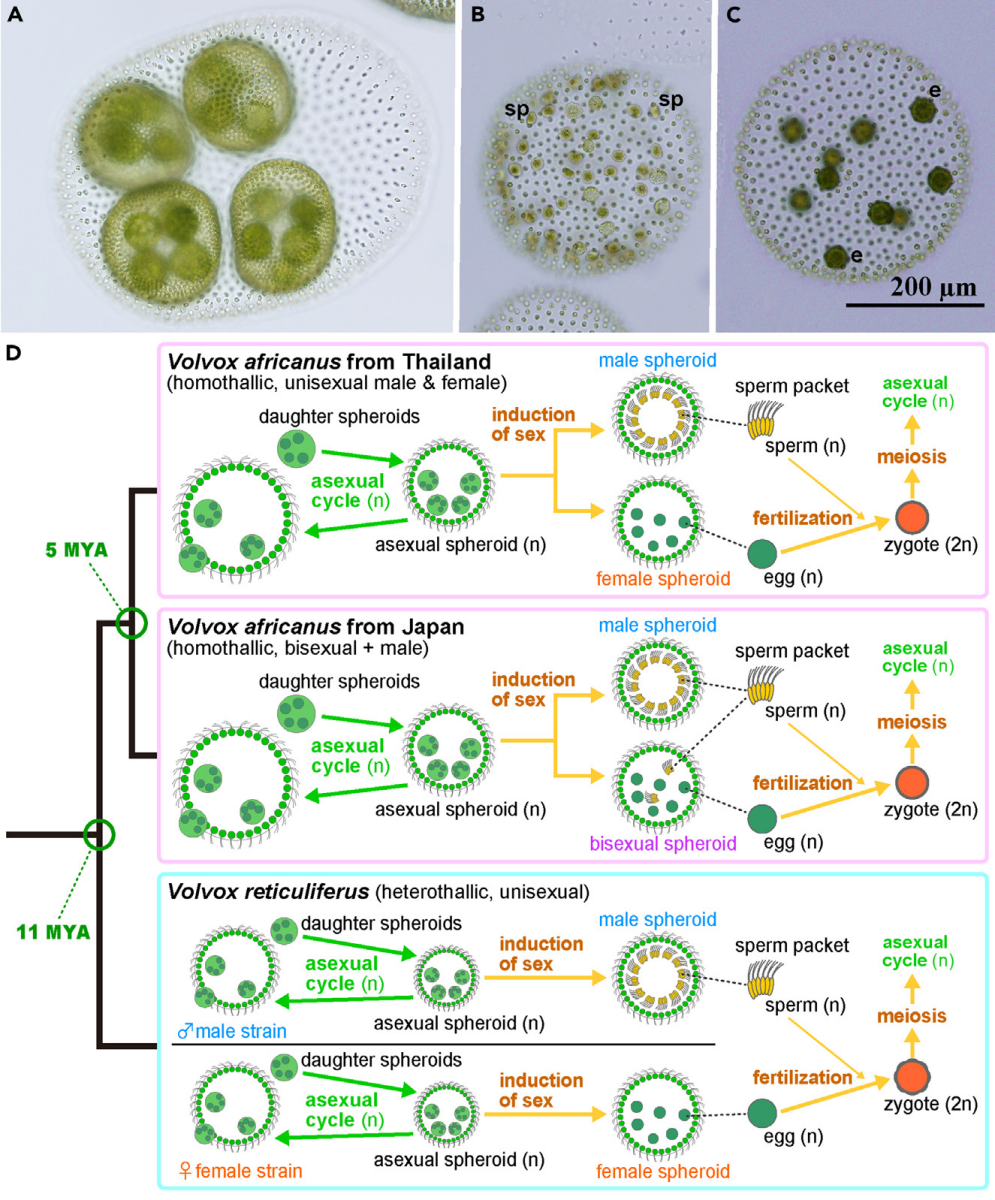
Two homothallic mating systems of *Volvox africanus* and heterothallic *V. reticuliferus* (A–C) Light microscopy of *V. africanus* with a homothallic mating system of male-female type (Table S1) originating from Thailand,^7^ shown at the same magnification. (A) Asexual spheroid. (B) Male spheroid with sperm packets (sp). (C) Female spheroid with eggs (e). (D) Schematic representation of phylogeny and life cycles of three mating systems of *Volvox africanus* and *V. reticuliferus*, subjected to the present genome comparison. Based on Yamamoto et al.^4^ and Nozaki et al.^7^

During a field survey in the interior of Thailand, culture strains of *V. africanus* that had a different homothallic mating system (homothallic, male-female type) from that of *V. africanus* JP previously examined were obtained^4^^,^^7^ (Figure 1 and Table S1). Therefore, this study examined the fate of the SDR of the ancestral heterothallic species during the evolution of the homothallic mating systems in *V. africanus* originating from Thailand, based on *de novo* whole-genome sequencing.

## Results and discussion

### Whole-genome assembly

A *de novo* nuclear genome of *V. africanus* strain 1101-NZ-11 originating from Thailand (*V. africanus* TH)^7^ was constructed by assembling a combination of long and short sequencing reads (see STAR Methods) and constituted 129 contigs. The 129 contigs were gap free (N50 = 3.95 Mbp) and yielded a nuclear genome assembly of 141.0 Mbp, similar to that of *V. carteri* and other volvocine species (Tables S2 and S3). Protein-coding gene predictions were performed with the assistance of transcriptome data to estimate 13,455 expressed genes in the genome (Table S2). Assembly quality of the genome was high based on the presence of the vast majority of benchmarking universal single-copy orthologs (BUSCO) reference genes^8^ (98.1% complete genes) (Table S2). In addition, the genome size estimated based on k-mer frequencies (Figure S1) using GenomeScope^9^ was consistent with the total genome assembly size (Table S2), suggesting high coverage rates of the assembled contigs for the whole genome.

### An expanded SDLR resolved

**Figure 2:**
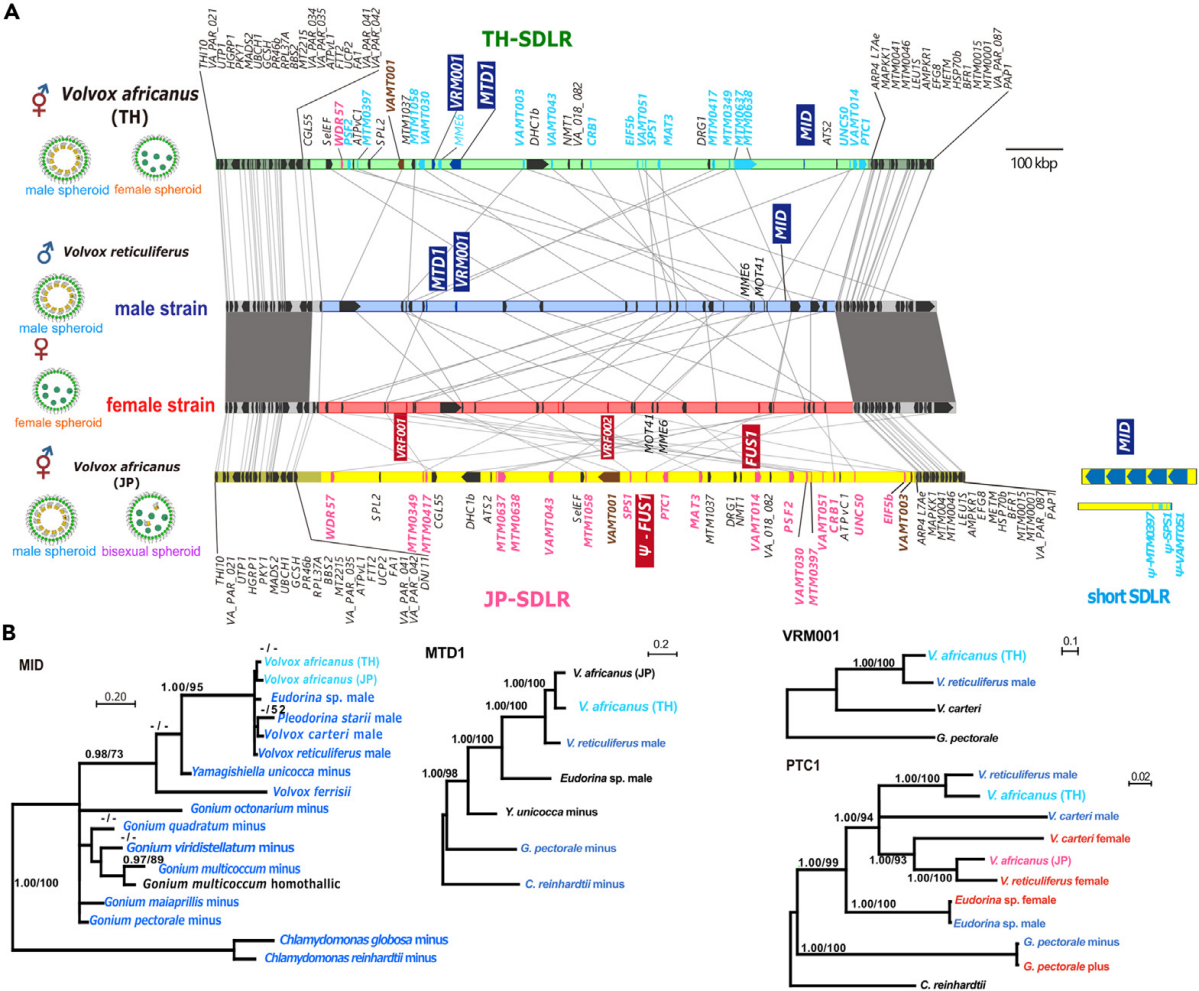
Sex-determining region (SDR) of heterothallic *Volvox reticuliferus* (male and female) and sex-determining-like regions (SDLRs) of two homothallic mating systems (Thai [TH] and Japanese [JP]) in homothallic *V. africanus* and phylogeny of three genes in SDR and SDLR (A) Comparison of SDLRs from two homothallic mating systems of *V. africanus* (TH-SDLR [accession LC749599] from Thai culture strain, and JP-SDLR [accession LC586641] and short SDLR [LC586642] from Japanese culture strain) and *V. reticuliferus* male and female SDR (accession LC586643 through LC586644). Note male- and female-specific genes (with blue or red backgrounds, respectively) and gametologs of *V. reticuliferus* and their homologs in SDLRs of *V. africanus.* Red and blue regions represent female and male SDRs, respectively. Green and yellow regions represent TH-SDLR and JP-SDLR/short SDLR, respectively. Gray shading indicates a syntenic bloc of pseudo autosomal regions. Red and blue homologs in SDLRs represent close relationships to female and male *V. reticuliferus* gametologs, respectively (deduced from phylogenetic analyses; (B) and Figures S5–S7). (B) Phylogenetic trees based on Bayesian inference (BI) using homologs of male-specific *MID*, *MTD1*, and *VRM001*, and gametolog *PTC1*. Red and blue represent homologs of fully sex-linked genes from female (mating type plus) SDR (including JP-SDLR) and male (mating type minus) SDR (including TH-SDLR and short SDLR), respectively. Numbers in left and right sides at branches indicate posterior probabilities (0.90 or more) of BI and bootstrap values (50% or more) of maximum likelihood analysis, respectively. For phylogenetic analyses of other genes in TH-SDLR, refer to Figures S5–S8.

Based on a genome search using the two contigs harboring male and female SDRs in *V*. *reticuliferus* and the contig harboring SDLR (JP-SDLR) in *V*. *africanus* JP,^4^ we found a homologous *V*. *africanus* TH contig (contig0022), which had two separate sequences corresponding to the two separate sequences flanking JP-SDLR, as well as to the two pseudo autosomal regions flanking SDR of *V*. *reticuliferus*^4^ (Figure S2). A long ca. 1 Mbp sequence (TH-SDLR) was found between the two separate sequences in contig0022 of *V*. *africanus* TH (Figure 2A). TH-SDLR had no dot-plot similarity to the SDRs of *V*. *reticuliferus* or JP-SDLR (Figures S2–S4), suggesting rapid movement in positions of the genes (gametologs in SDR^10^^,^^11^ and their homologs in SDLR^4^) during evolution. However, it was repeat rich and had a lower GC content than those of the whole-genome sequences found in SDRs of heterothallic volvocine species and JP-SDLR (Table S3).

**Figure 3:**
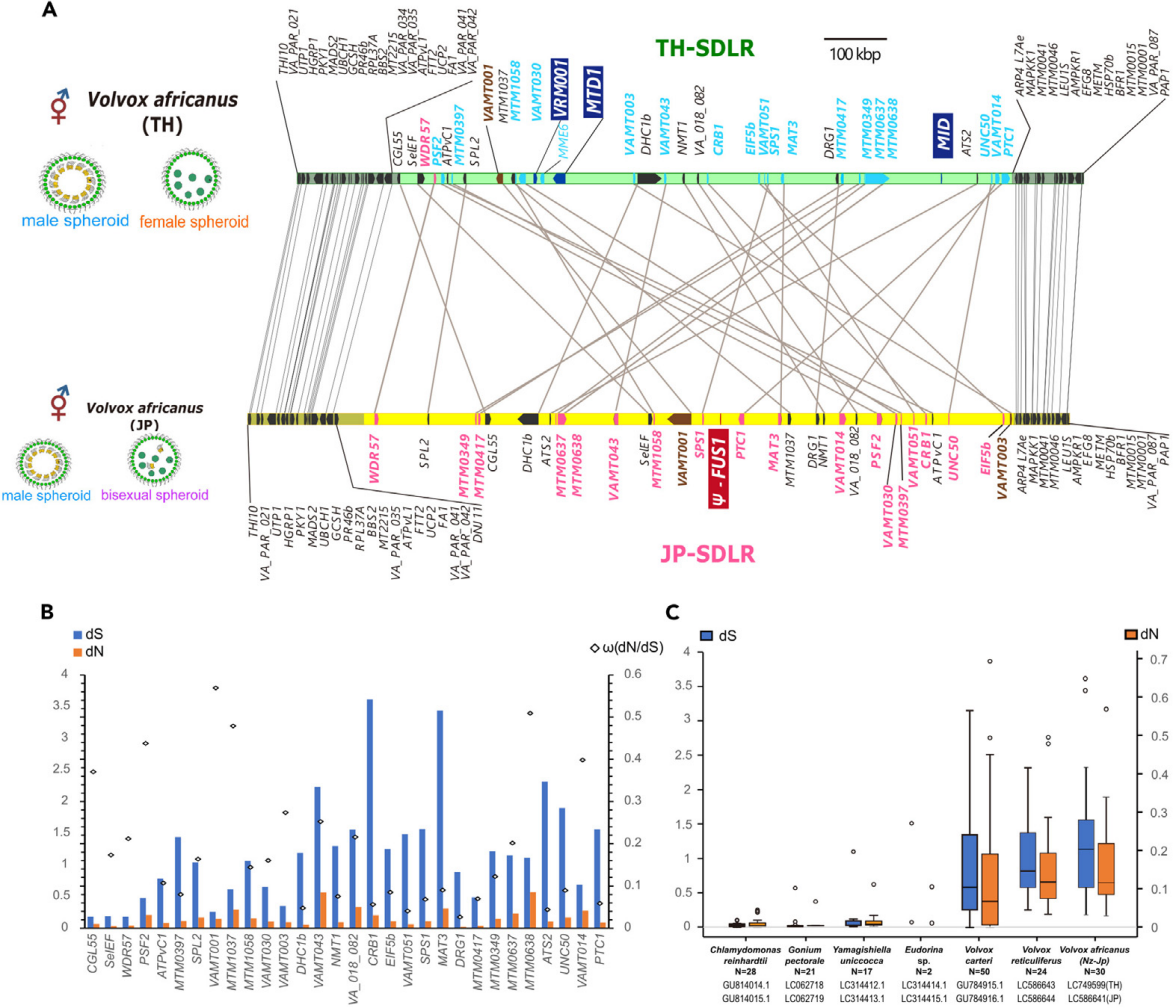
Comparison between two sex-determining-like regions of Thai and Japanese strains (TH-SDLR and JP-SDLR, respectively) of homothallic *Volvox africanus* (A) Comparison between TH-SDLR and JP-SDLR, showing ancestral male and female SDRs, respectively, Green and yellow regions represent TH-SDLR and JP-SDLR, respectively. Gray shading indicates a syntenic bloc of pseudo autosomal regions. Red and blue homologs in SDLRs represent close relationships to female and male *V. reticuliferus* gametologs, respectively (deduced from phylogenetic analyses; Figures 2B and S5–S7). (B and C) Molecular evolutionary analyses of 30 gametolog-like genes shared by TH-SDLR and JP-SDLR. (B) Synonymous (dS, blue/left) and non-synonymous (dN, orange/right) substitution values between homologous genes in TH-SDLR and JP-SDLR. There are no prominently dimorphic pairs under positive selection between SDLRs (dN/dS > 1). (C) Box-whisker plots comparing the distributions of dS (blue) and dN (orange) substitution values for gametolog-like gene pairs found in SDRs of volvocine algal haploid UV chromosomes and SDLRs of homothallic *V. africanus*. Open dots are outliers from interquartile ranges except for those of *Eudorina* sp., which indicate two gametologs.

TH-SDLR harbored homologs of all three male-specific genes in *V*. *reticuliferus* (*MID*, *MTD1*, and *VRM-001*) and 31 other protein-coding genes (Figure 2A); 21 of the 31 were homologs for *V*. *reticuliferus* gametologs^4^ (Figures 2B and S5–S7 and Table S4), whereas seven of the other 10 were homologs of *V*. *carteri* gametologs^10^ (Figure S8). Interestingly, 30 of the 31 genes were homologous between TH-SDLR and JP-SDLR, and their divergences were similar to those of gametolog pairs in heterothallic volvocine species (Figure 3); the one remaining gene was a homolog of *MME6* that is recognized in the SDR of *V*. *reticuliferus* as a gametolog but is positioned in an autosome-like region (outside JP-SDLR) in *V*. *africanus* JP^4^ (Figures 2A and S7, and Table S4). Of the 30 genes shared between TH-SDLR and JP-SDLR, 20 were homologs of *V*. *reticuliferus* gametologs (Figures 2A, S5 and S6, and Table S4). However, TH-SDLR differed significantly from JP-SDLR in the origins of the homologs of the *V*. *reticuliferus* gametologs. Although 18 of the 20 homologs for *V*. *reticuliferus* gametologs in JP-SDLR originated from the female SDR of the ancestral heterothallic species and no male-related homologs were found in JP-SDLR,^4^ 18 of the 20 shared homologs and *MME6* in TH-SDLR were shown to have originated from the ancestral male SDR based on a phylogenetic analysis, while the remaining two (*WDR57* and *VAMT001*) had no resolution or female origin (Figures 2B and S5–S7). Therefore, TH-SDLR might have evolved from the male SDR of the ancestral heterothallic species.

### Putative ancestral heterothallic species of homothallic *V. africanus*

As discussed above, this study clearly demonstrated that TH-SDLR could have originated from the male SDR of the ancestral heterothallic species. By contrast, JP-SDLR might be female SDR derived.^4^ Since TH-SDLR and JP-SDLR shared 30 homologous genes that exhibit some divergence and different sex origins as the gametolog pairs in the heterothallic volvocine species (Figures 3, S5, S6, S8, and S9), these two SDLRs may have maintained the gene compositions of the male and female SDRs of the heterothallic ancestral species of *V*. *africanus*. The ancestral male and female SDRs might have harbored three male-specific genes (*MID*, *MTD1*, and *VRM-001*) and one female-specific gene (*FUS1*), respectively, and they might have had 30 gametologs (20 directly related to *V*. *reticuliferus* gametologs) (Figure S9). Therefore, the genomic features of the ancestral male and female SDRs (gene compositions, low GC content, and high repeat rate) might have been retained in TH-SDLR and JP-SDLR, respectively, in two different homothallic mating systems within homothallic *V*. *africanus* (Figures 1 and S9).

### Essential genes for homothallic mating system in *V. africanus*

A homothallic mating system is based on the presence of both male and female attributes of sexual reproduction in a single haploid genotype. Our current and previous studies clearly demonstrated that both of two different homothallic organisms (*V*. *africanus* TH and JP) have 30 possible ancestral gametologs in SDLR, while three male-specific genes (*MID*, *MTD1*, and *VRM001*) are located in TH-SDLR (Figure 2), or *MID* and *MTD1* are outside JP-SDLR (Figure S9).^4^ Therefore, maleness in the homothallic species is based on the presence of homologs of the conserved male-specific genes *MID* and *MTD1*.^4^^,^^10^^,^^11^ In comparison, the majority of the 30 possible ancestral gametologs in TH-SDLR and JP-SDLR are male or female related, respectively. Thus, irrespective of male or female SDR origin, the presence of the 30 ancestral gametologs in both SDLRs may be essential for sexual reproduction, especially the female-related attributes in homothallic *V*. *africanus* because the most important male-related attribute is the production of sperm packets that is determined by the male-specific gene *MID*.^12^ Retaining the ancestral genomic features of the SDR (low GC contents and repeat-rich; Tables S2 and S3) in both TH-SDLR and JP-SDLR may represent a very early event in the transition from heterothallism to homothallism in *V*. *africanus*. However, the continuous expanded (ca. 1 Mbp long) SDLRs and low GC and repeat-rich genomic features in SDLRs may be important for sexual reproduction of *V*. *africanus* because such expanded genome regions might have been retained in heterothallic species for at least 75 mya after the divergence between *V*. *carteri* and *V*. *reticuliferus*.^4^

### Possible evolutionary history of ancestral male and female SDRs during the evolution of the homothallism in *V. africanus*

The direct ancestral heterothallic species of *V*. *africanus* is thought to have male and female SDRs that are very similar to TH-SDLR and JP-SDLR, respectively (Figures 3 and S9). Given that the transition to homothallism occurred only once in *V*. *africanus* as suggested by the single origin of all the three homothallic mating systems within *V. africanus*,^6^ evolution from the heterothallic ancestor to the homothallic ancestor might have been initiated by the acquisition of both male and female SDRs of the ancestral heterothallic species (Figure S9). Subsequently, in the direct ancestor of *V*. *africanus* JP, the male SDR became degenerate, but the important male genes (*MID* and *MTD1*) and the female SDR were retained to express male and female functions in sexual reproduction. By contrast, the female SDR might have disappeared completely in the direct ancestor of *V*. *africanus* TH. Experimental transition from heterothallism to homothallism was demonstrated using partial suppression of the *MID* gene in the male strain of *V*. *carteri*.^12^ However, the important male attribute “sperm packet formation” can be induced in the female strain of *V. carteri* by transforming the male-specific gene *MID*.^12^ Thus, evolutionary transition from heterothallism to homothallism may be possible by using only the genes of the male genotype in the genus *Volvox*.

### Limitations of the study

Based on the present genome comparison, each of the expanded male and female ancestral SDRs may have been conserved during the evolution of the homothallic species *V. africanus*, and such an ancestral SDR or SDLR appears to be essential for homothallic sexual reproduction in *V. africanus*, regardless of male or female origin. The expanded SDLRs have low GC and repeat-rich genomic features (Table S2), which appear to be widely conserved in SDRs of heterothallic species of *Volvox* related to *V. carteri* and *V. reticuliferus*.^4^ SDRs are recognized in the marine unicellular species “prasinophytes” based on their low GC and repeat-rich contiguous genomic features even when sexuality is unknown.^13^ However, the molecular genetic relationships between these genomic features in SDR (or SDLR) and sexuality are unknown. Thus, further studies are needed to elucidate the biological significance of such expanded genomic regions. There will also be many interesting questions about the expanded SDR or SDLR in *Volvox*. Apart from the identified sex-linked genes, what are the possible important genes in determining the mating system and/or phenotype of a sexual spheroid (Table S1)? Is the genome structure important in projecting further genome arrangements in SDR/SDLR? Is the epigenome involved in SDR/SDLR?

## STAR★Methods

### Key resources table

**Table:** 

REAGENT or RESOURCE	SOURCE	IDENTIFIER
**Biological samples**		

*Volvox africanus* TH (strain 1101-NZ-11)	Nozaki et al.^7^	NIES-4468

**Chemicals, peptides, and recombinant proteins**		

TURBO DNA-*free* Kit	Thermo Fisher Scientific	Cat#AM1907

**Critical commercial assays**		

NucleoBond HMW DNA	Macherey-Nagel	Cat#740160.20
TruSeq DNA PCR-Free Sample Prep Kit	Illumina	Cat#20015962
Zymoclean Large Fragment DNA Recovery Kit	Zymo Research	Cat#D4045
RNeasy Plant Mini Kit	Qiagen	Cat#74904
TruSeq Stranded mRNA Library Prep Kit	Illumina	Cat#20020594

**Deposited data**		

*Volvox africanus* TH sequence reads	This paper	DRA: DRA015422
*Volvox africanus* TH assembly	This paper	DDBJ: BSDZ01000001–BSDZ01000129
*Volvox africanus* TH sex-determining-like region sequence	This paper	DDBJ: LC749599
*Volvox africanus* JP sex-determining-like region sequence	Yamamoto et al.^4^	DDBJ: LC586641
*Volvox africanus* JP short sex-determining-like region sequence	Yamamoto et al.^4^	DDBJ: LC586642
*Volvox reticuliferus* female sex-determining region	Yamamoto et al.^4^	DDBJ: LC586643
*Volvox reticuliferus* male sex-determining region	Yamamoto et al.^4^	DDBJ: LC586644
Alignments used for molecular phylogenetic analyses	This paper	TreeBASE: S29849

**Software and algorithms**		

BUSCO v5.1.2	Manni et al.^8^	https://busco.ezlab.org/
GenomeScope	Vurture et al.^9^	https://github.com/schatzlab/genomescope
Canu v2.2	Koren et al.^14^	https://github.com/marbl/canu
Pilon v1.23	Walker et al.^15^	https://github.com/broadinstitute/pilon
YASS	Noé et al.^16^	https://github.com/laurentnoe/yass
HISAT v2.2.1.	Pertea et al.^17^	https://github.com/DaehwanKimLab/hisat2
StringTie v2.2.1	Pertea et al.^17^	https://github.com/gpertea/stringtie
TransDecoder v5.5.0	GitHub	https://github.com/TransDecoder/TransDecoder/releases
MEGA X 10.0.5	Kumar et al.^18^	https://www.megasoftware.net/
trimAl 1.2	Capella-Gutiérrez et al.^19^	https://github.com/inab/trimal
MrBayes 3.2.7	Ronquist et al.^20^	https://github.com/NBISweden/MrBayes
RAxML-NG 0.9	Kozlov et al.^21^	https://github.com/amkozlov/raxml-ng
ModelTest-NG 0.1.6	Darriba et al.^22^	https://github.com/ddarriba/modeltest
PAML 4.9i	Yang^23^	https://github.com/abacus-gene/paml

### Resource availability

#### Lead contact

Further information and requests for resources and reagents should be directed to and will be fulfilled by the lead contact, Hisayoshi Nozaki (hisayoshi.nozaki@gmail.com).

#### Materials availability

This study did not generate new unique reagents.

### Experimental model and subject details

The Thai culture strain of *V. africanus* strain 1101-NZ-11 (=NIES-4468)^7^ (*V. africanus* TH) was used. The culture was maintained in screw-cap tubes (18 × 150-mm) containing 10 mL Volvox thiamin acetate (VTAC) medium,^24^ at 25 °C under a 14:10-h light:dark schedule, under cool-white fluorescent lamps at an intensity of 80–130 μmol m^−2^ s^−1^. Analysis of Thai materials was conducted in accordance with a Memorandum of Understanding between the University of Tokyo and Khon Kaen University for international cooperative research on the systematics, phylogenetics, and evolution of freshwater green algae in Thailand (2017–2021).

### Method details

#### Whole-genome sequencing and *de novo* assembly

Cultures of *V. africanus* TH were grown in Petri dishes (100 mm/non-treated Dish, IWAKI AGC Techno Glass, Shizuoka, Japan) containing 30 mL VTAC medium at 25°C on a 14:10 h light: dark (L:D) schedule, under cool-white fluorescent lamps at an intensity of 80–130 μmol m^−2^ s^−1^. Approximately 200 mL cultured sample was subjected to genomic DNA extraction. Genomic DNA was prepared by using NucleoBond HMW DNA (Macherey-Nagel, Düren, Germany) according to the manufacture’s protocol. A whole-genome sequencings of *V. africanus* TH was performed using PacBio and Illumina technologies. Genomic DNA was sheared using a DNA shearing tube, g-TUBE (Covaris). Two sequencing libraries (20 kbp and 30 kbp) were constructed and each library was sequenced on one single molecule, real-time (SMRT) cell using the PacBio Sequel system (Pacific Biosciences, Menlo Park, CA, United States). These reactions generated 1.48 M subreads (total bases: 27.7 Gbp). Sequencing coverage was about 197x based on the estimated genome size. The PacBio reads were assembled *de novo* with Canu v2.2.^14^ Furthermore, genomic DNA was fragmented with a DNA Shearing System, S2 Focused-ultrasonicator (Covaris ILC, Woburn, MA, United States). Illumina paired-end library (average insert size 520 bp) was constructed with a TruSeq DNA PCR-Free Sample Prep Kit (Illumina, San Diego, CA, United States) according to the manufacturer’s instructions and were size-selected on an agarose gel using a Zymoclean Large Fragment DNA Recovery Kit (Zymo Research, Irvine, CA, United States). The final library was sequenced on the Illumina NovaSeq 6000 sequencer (156 M reads with 150 bp read length). Total bases and sequence coverage were 23.5 Gbp and 166-fold, respectively. The Illumina data were then mapped against the PacBio assembly sequences, and assemblies were corrected using Pilon v1.23.^15^ Contigs with ≦2% or ≧98% GC, with ≧10% matching with bacterial or organelle DNA sequences, or with ≦10x mean coverage of Illumina reads were excluded from a set of the nuclear genome sequences. The completeness of the genome assembly was verified using the BUSCO v5.1.2^8^ with 1,519 single-copy orthologs from the chlorophyta_odb10 dataset, which indicated that 98.1% complete genes in the reference dataset were present in the current genome assembly (Table S2).

#### SDLR identification

A candidate contig (contig022) for the entire SDLR was screened as major significant matching subjects with more than three nonoverlapping protein hits (cutoff maximum E-value: 1 × 10−10) by TBLASTN (National Center for Biotechnology Information) on *de novo* genome assemblies of *V. africanus* TH with all proteins on *V. reticuliferus* male SDR and female SDR as queries and then dotplot analyses were performed between contig022 of *V. africanus* TH and *V. reticuliferus* male SDR using YASS (https://bioinfo.lifl.fr/yass/index.php)^16^ to detect the SDLR. By using sequences of the *V. reticuliferus* SDR and SDLR of the Japanese homothallic strain of *V. africanus* (JP-SDLR),^4^ a long SDLR (TH-SDLR) was determined in contig022 of *V. africanus* TH.

#### Gene identification

We performed TBLASTN searches against the genome assembly databases of *V. africanus* TH genotype with the volvocine fully sex-linked (sex-specific) gene proteins as the queries, retrieved sequences with the highest similarity. Other gene models on TH-SDLR and on autosomal/autosome-like regions were constructed manually after predicted by RNA sequencing mapping. For RNA sequencing mapping, total RNA was extracted from approximately 200 mL culture using RNeasy Plant Mini Kit (Qiagen, Hilden, Germany) according to the manufacturer’s protocol. Contaminating DNA was removed using TURBO DNA-*free* Kit (Thermo Fisher Scientific, MA, USA). The RNA-seq libraries were constructed using a TruSeq Stranded mRNA Library Prep (Illumina, San Diego, CA, United States) and were sequenced on the Illumina NovaSeq 6000 instruments. The Illumina reads from each sample were mapped to the reference genome with HISAT v2.2.1,^17^ setting the parameters of “-q --phred33 -p 16 --rna-strandness RF”. The mapped reads were then assembled into transcripts by StringTie v2.2.1^17^ with “--rf” option. The candidate coding regions in transcript sequences were then predicted by TransDecoder v5.5.0 (https://github.com/TransDecoder/TransDecoder/releases) with “-S″ option for Transdecoder.LongOrfs and “--retain_blastp_hits" option for Transdecoder.Predict (Database: TrEMBL).

#### Molecular phylogenetic analyses

Homologous protein sequences of fully sex-linked genes and gametologs in *V. africanus* TH were retrieved from database of other volvocine algae by BLASTP on National Center for Biotechnology Information. Sequences were aligned using MUSCLE on MEGA X 10.0.5,^18^ and ambiguous sites in respective matrices were automatically removed using trimAl 1.2^19^ with the “gappyout” option. For each matrix, a molecular phylogenetic analysis based on Bayesian inference was conducted using MrBayes 3.2.7.^20^ One million generations of Markov chain Monte Carlo iterations were carried out, and the first 25% were discarded as burn-in. The average standard deviation of split frequencies was below 0.01, indicating convergence of the analysis. The maximum likelihood analysis using RAxML-NG 0.9^21^ was also performed for respective matrices, to estimate the bootstrap values^25^ based on 1,000 replications. The best-fitted models selected by ModelTest-NG 0.1.6^22^ are shown in Table S5. The alignments used for the analyses are available in TreeBASE (https://www.treebase.org/treebase-web/home.html; Study ID, S29849).

### Quantification and statistical analysis

Divergence scores of synonymous and non-synonymous substitutions between homologous genes were computed using yn00 of the PAML 4.9i^23^; synonymous and non-synonymous substitution rates of aligned coding sequences were estimated based on the method of Yang and Nielsen^26^ with equal weighting and the same codon frequency for all pairs.

## Data Availability

New genome and RNA-seq data have been deposited at DNA DataBank of Japan (DDBJ)/European Nucleotide Archive (ENA)/GenBank and are publicly available as of the date of publication. Accession numbers are listed in the key resources table. All other study data are included in the article and/or Supplemental information. This paper does not report original code. Any additional information required to reanalyze the data reported in this paper is available from the lead contact upon request.
