# Supplementary material for: Expanded male sex-determining region conserved during the evolution of homothallism in the green alga Volvox
Source: iScience. 2023 Jun 1;26(6):106893. doi: 10.1016/j.isci.2023.106893 (PMC10291315; doi:10.1016/j.isci.2023.106893)
Supplement: Document S1. Figures S1–S9 and Tables S1–S5 [file mmc1.pdf]

iScience, Volume *26*

Supplemental information

Expanded male sex-determining region conserved during the evolution of homothallism in the green alga *Volvox*

Kayoko Yamamoto, Ryo Matsuzaki, Wuttipong Mahakham, Wirawan Heman, Hiroyuki Sekimoto, Masanobu Kawachi, Yohei Minakuchi, Atsushi Toyoda, and Hisayoshi Nozaki

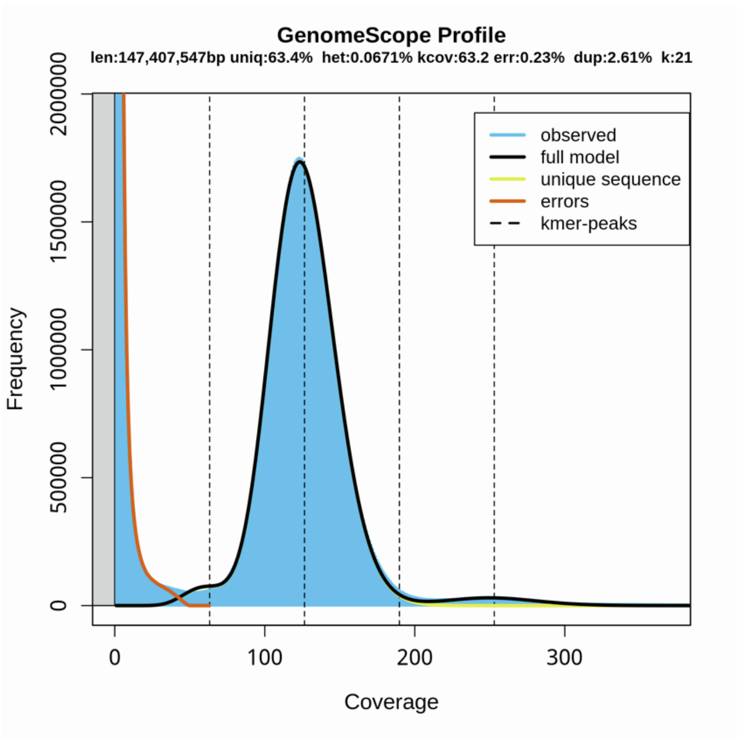

Figure S1. GenomeScope[1] profile of *Volvox africanus* strain 1101-NZ-11 (TH), Related to Star Methods. Based on the unmapped reads to *Volvox africanus* organelle genome sequences.

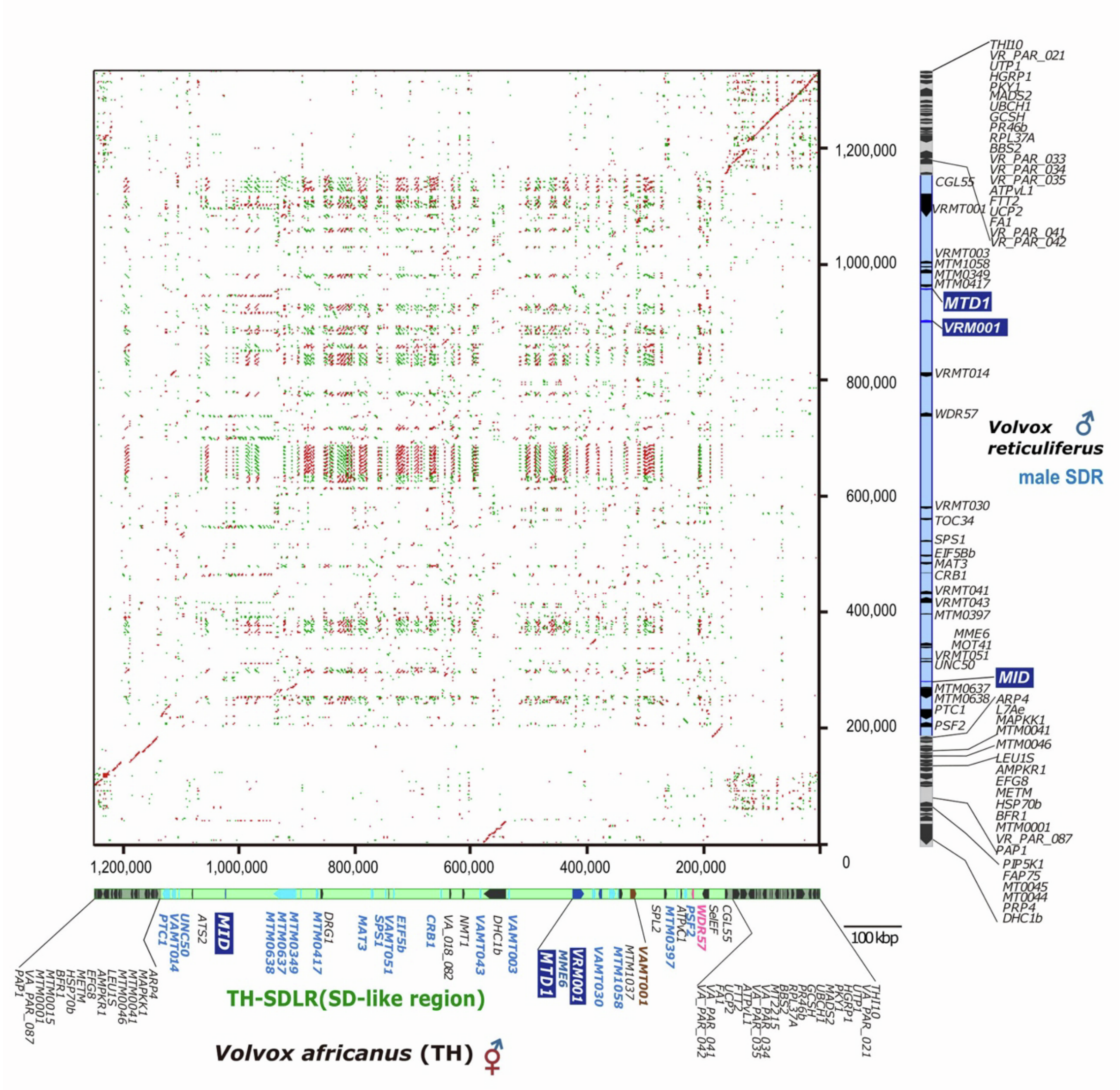

Figure S2. Dotplot between male sex-determining region (SDR) of heterothallic *Volvox reticuliferus* (vertical) and its homologous sex-determining-like region (SDLR) of Thai culture strain of homothallic *V. africanus* (horizontal) and parts of flanking or pseudo autosomal regions (gray), Related to Figure 2A. Green and blue dots indicate forward and reverse alignments, respectively. For details of SDR and SDLR, see Figure 2A and Yamamoto et al.[2]

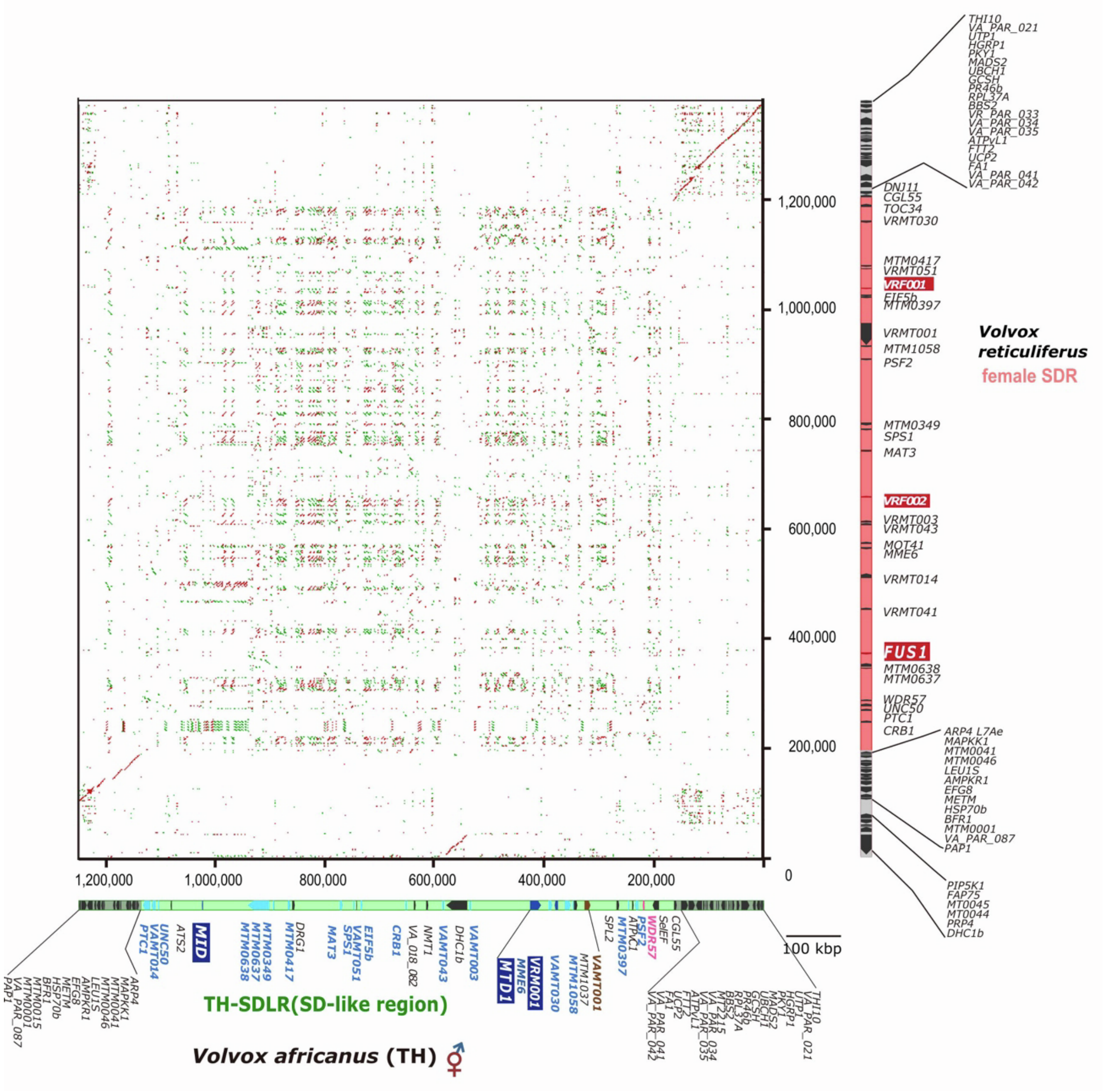

Figure S3. Dotplot between female sex-determining region (SDR) of heterothallic *Volvox reticuliferus* (vertical) and its homologous sex-determining-like region (SDLR) of Thai culture strain of homothallic *V. africanus* (horizontal) and parts of flanking or pseudo autosomal regions (gray), Related to Figure 2A. Green and blue dots indicate forward and reverse alignments, respectively. For details of SDR and SDLR, see Figure 2A and Yamamoto et al.[2]

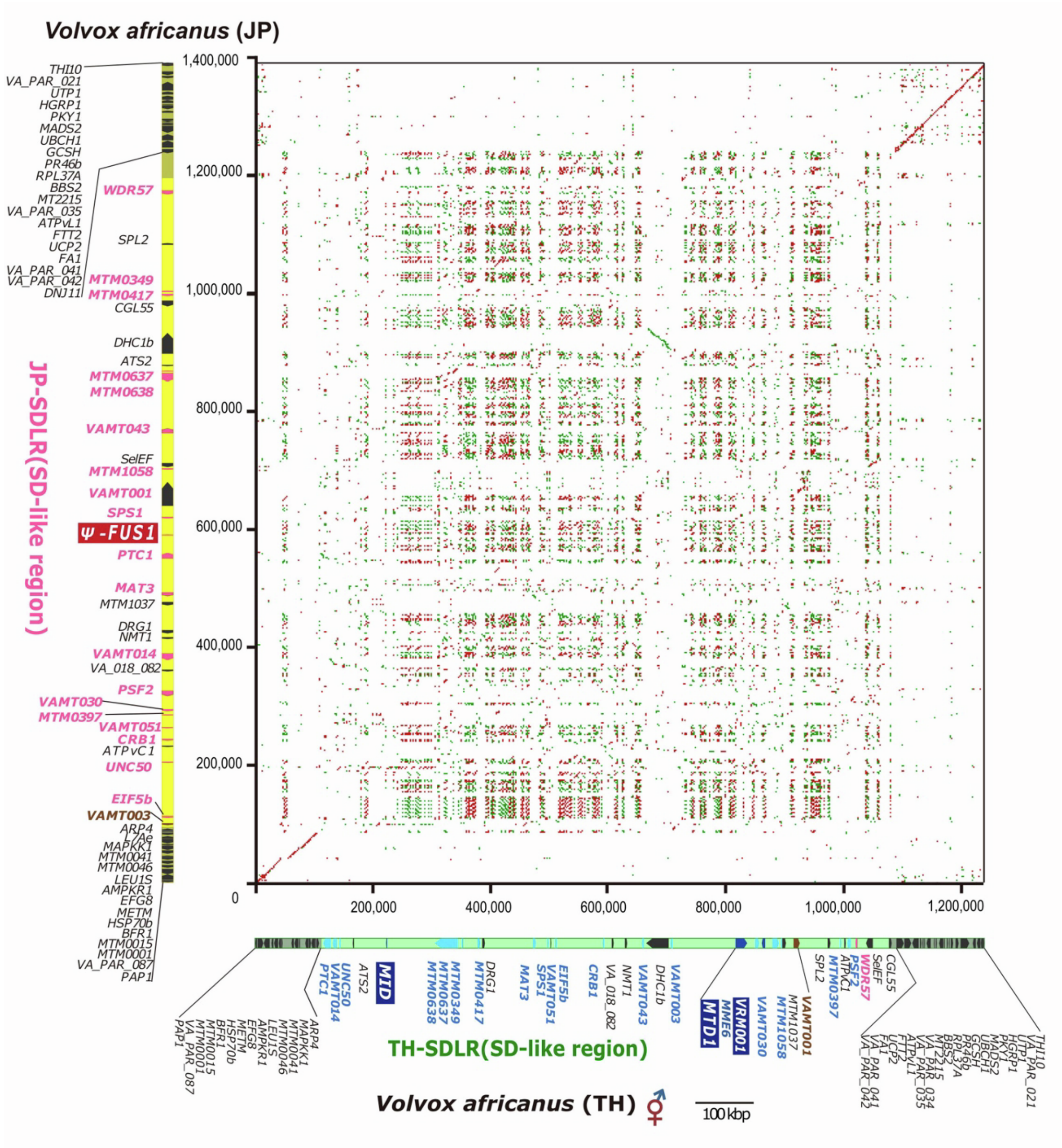

Figure S4. Dotplot between sex-determining-like regions (SDLRs) in culture strains from Thai (vertical) and Japan (longitudinal) in homothallic *Volvox africanus*, Related to Figure 2A. Note each SDLR (green vertical bar in green or yellow longitudinal bar) and parts of flanking regions (gray) that are homologous to pseudo autosomal regions of heterothallic *V. reticuliferus* (Figures 2A and S2). Green and blue dots indicate forward and reverse alignments, respectively. For details of SDLR, see Figure 2A and Yamamoto et al.[2]

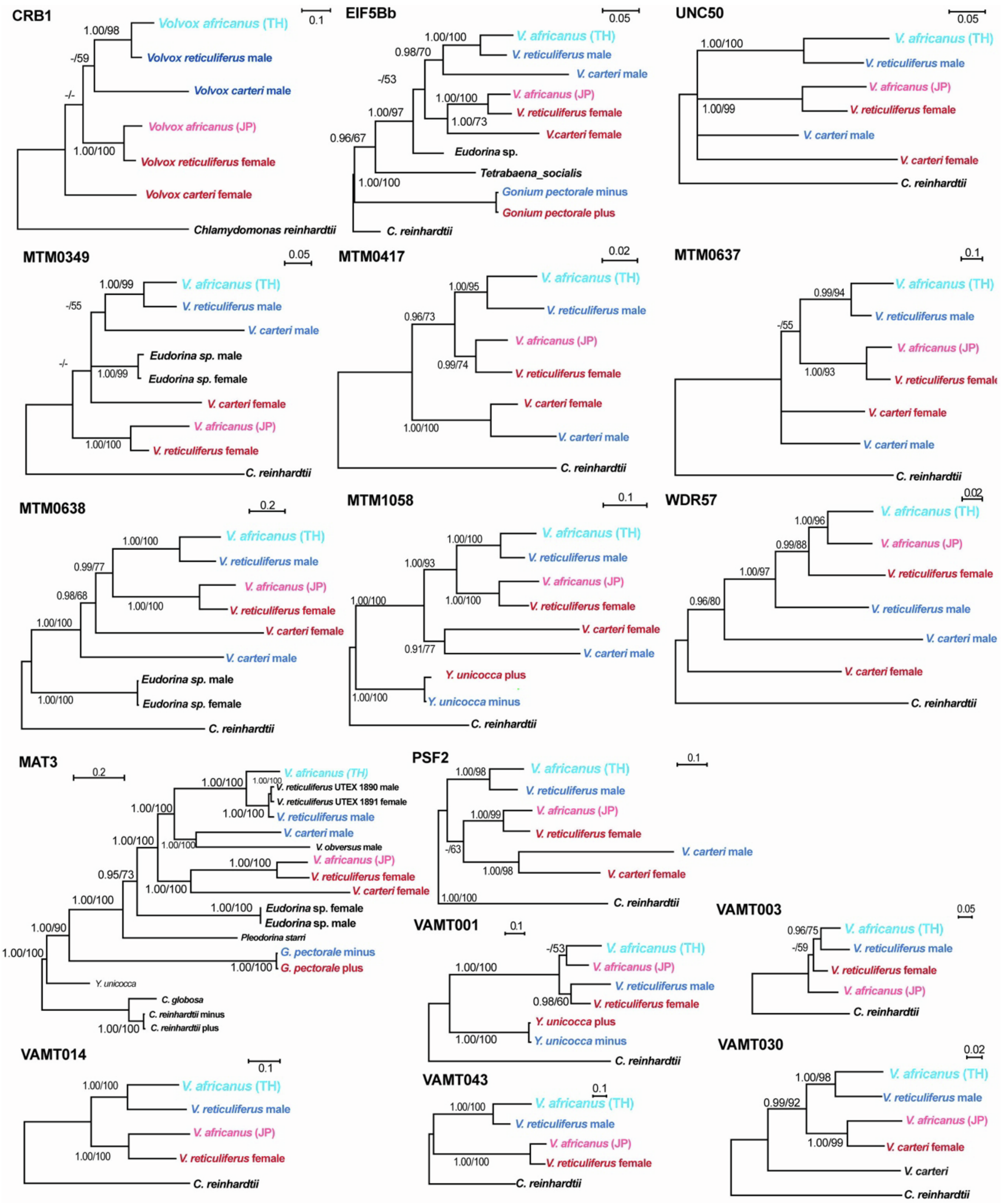

Figure S5. Bayesian inference (BI) phylogenetic trees of 16 of 20 TH-SDLR genes, Related to Figure 2A. The 16 are homologs of gametologs of heterothallic *Volvox reticuliferus*.[2] Red and blue represent homologs of fully sex-linked genes from female (mating type plus) SDR (including JP-SDLR) and male (mating type minus) SDR (including TH-SDLR and short SDLR), respectively. Numbers in left and right sides at branches indicate posterior probabilities (0.90 or more) of BI and bootstrap values (50% or more) of maximum likelihood analysis, respectively. For phylogenetic analyses of the other four genes, refer to Figures 2B and S6.

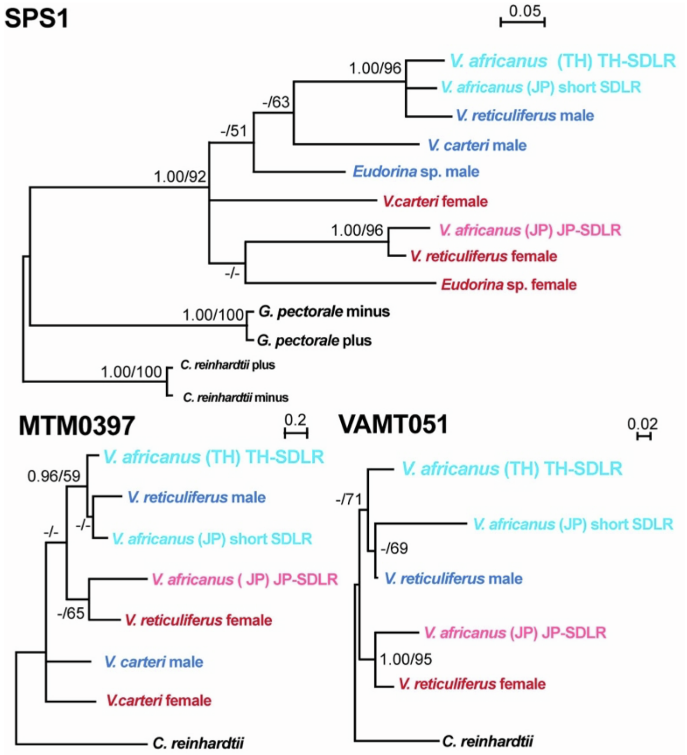

Figure S6. Bayesian phylogeny of three of 20 TH-SDLR genes, Related to Figure 2. The three genes are homologs of gametologs of heterothallic *Volvox reticuliferus*.[2] Note that each of these genes has homologs of both long SDLR (JP-SDLR) and short SDLR of homothallic *V. africanus* from Japan.[2] For more details, refer to the legend of Figure S5.

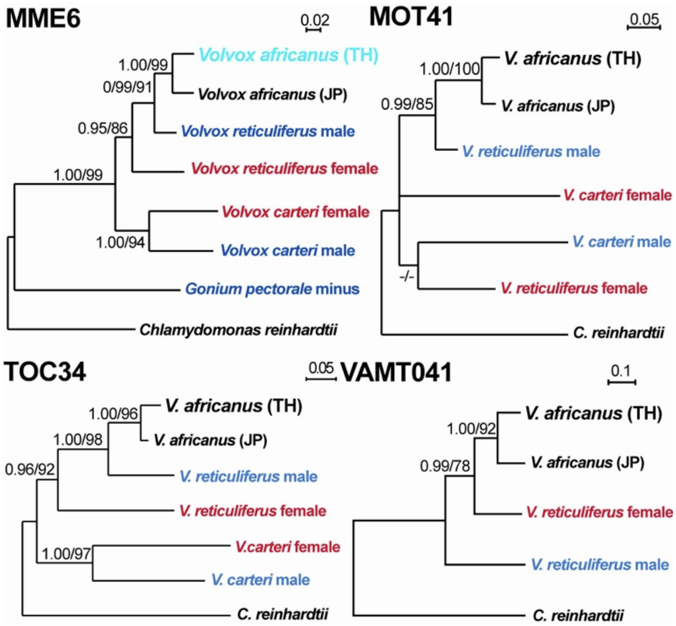

Figure S7. Bayesian phylogeny of four homologs of 24 gametologs of heterothallic *Volvox reticuliferus*,[2] Related to Figure 2. Note that each tree has one or two homologs that are localized in the autosome-like regions (black, outside SDLRs) of homothallic *V. africanus* from Japan[2] and Thai (Figure 2A). For more details, refer to the legend of Figure S5.

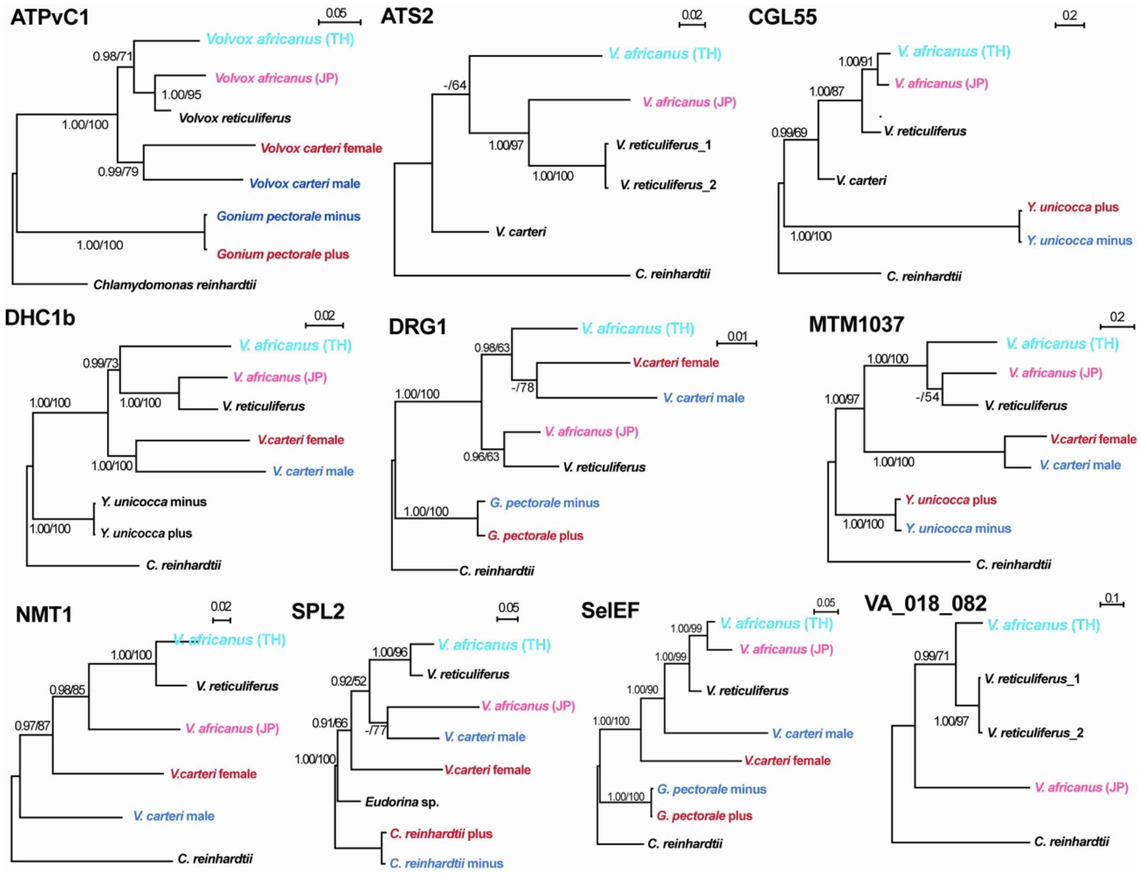

Figure S8. Bayesian phylogeny of 10 homologous pairs of SDLR genes, Related to Figure 3. The 10 pairs are shared between Thai (TH) and Japanese (JP) culture strains of homothallic *Volvox africanus* but lack homologous gametologs in heterothallic *V. reticuliferus*[2] (Figures 2A and 3A). For more details, refer to the legend of Figure S5.

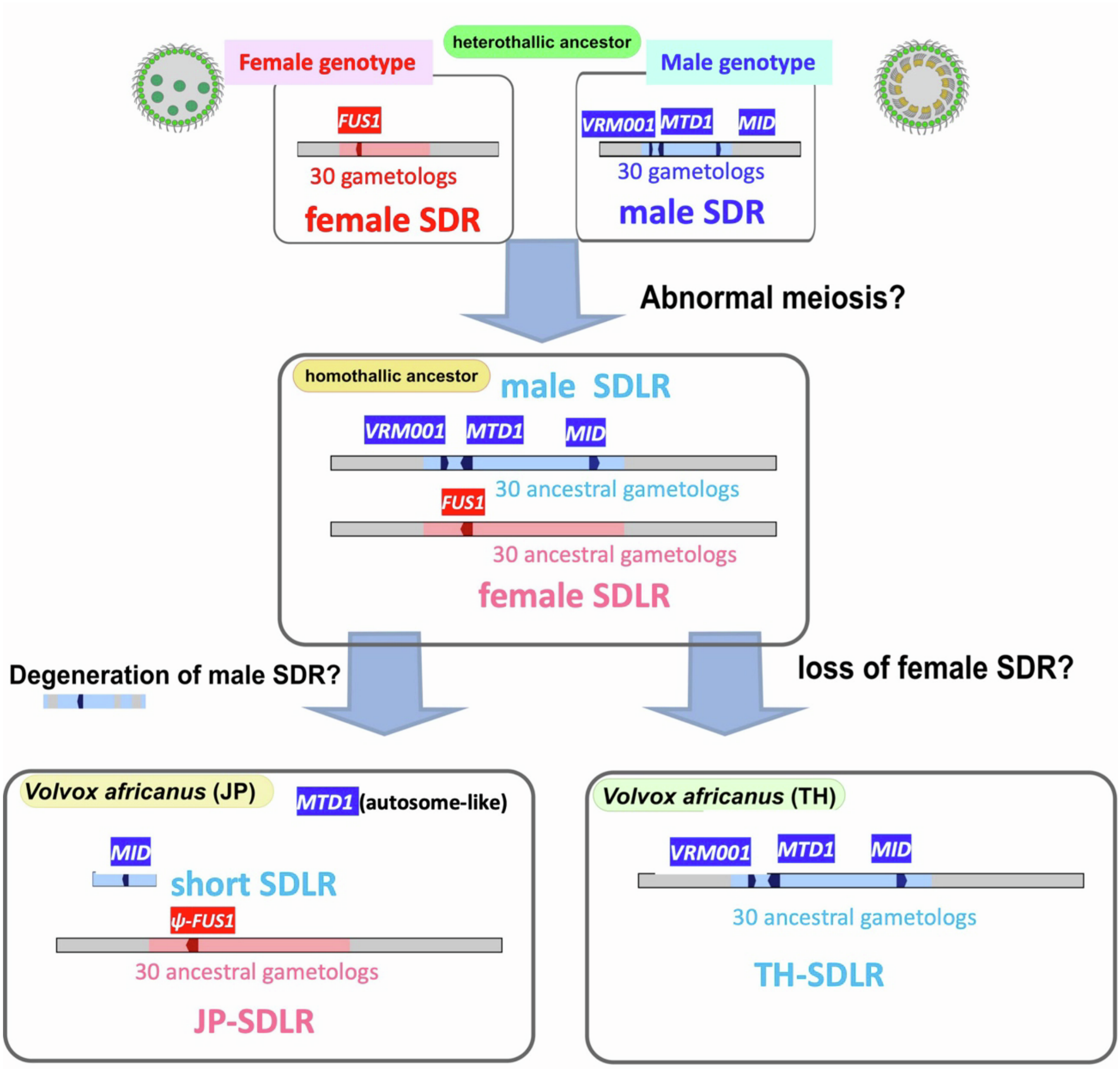

Figure S9. Hypothetical processes of evolution of two homothallic mating systems in *Volvox africanus* (JP and TH), Related to Figures 1-3. Note transition from sex-determining regions (SDRs) in ancestral heterothallic species to two types of sex-determining-like regions (SDLRs) in the two homothallic mating systems (JP and TH) (Figure 1 and Table S1). Based on the present whole genome comparison.

Table S1. Four types of mating systems in two closely related green algal species *Volvox africanus* and *V. reticuliferus*, Related to Figure 1.

Mating system	heterothallic type	homothallic, male-female type	homothallic, male-bisexual type	homothallic, bisexual type
Basic sexuality	heterothallism*	homothallism**	homothallism**	homothallism**
Sexual spheroids induced***	male or female spheroids in a single genotype	both male and female spheroids in a single genotype	both male and bisexual spheroids in a single genotype	only bisexual spheroid
Volvox species	*V. reticuliferus*	*V. africanus*	*V. africanus*	*V. africanus*
References	Starr[3], Nozaki et al.[4]	Starr[3], Nozaki et al.[5]	Starr[3], Nozaki et al.[4]	Starr[3]

*Genetically self-incompatible mating system, in which male or female is determined by the two complementary genotypes and a single clonal culture produces only one type of gametes, eggs in female spheroids or sperm in male spheroids. Thus, male or female is determined genetically in the heterothallic species. The heterothallic species perform only outcrossing.

**Genetically self-compatible mating system, in which gametes of both sexes (eggs and sperm) are produced within a single clonal culture (the same genotype). Thus, the homothallic species have possibility to form zygotes between gametes from identical genotype or a single clonal culture (selfing).

****Volvox* sexual spheroids (individuals) may be “unisexual” (male or female) or “bisexual” (production of both sperm and eggs) depending upon a species or lineage. Thus, the homothallic *Volvox* mating system is further classified into three subtypes based on production of the types of sexual spheroids.[3]

Table S2. Comparison of details of whole nuclear genomes and SDR or SDLR⁄short SDLR of four culture strains of two closely related species, *Volvox reticuliferus* and *V. africanus,* Related to Figure 1 and Star Methods.

	Species	*Volvox reticuliferus*		*Volvox africanus*	
	Mating type/ sex (culture strain)	Female (NIES-3786)	Male (NIES-3785)	Homothallic (Japanese NIES-3780)	Homothallic (Thai 1101-NZ-11)
Whole genome	Total length (bp) [Total number of gaps/non-ATGC nucleotides (bp)]	133,065,142 [0/0]	133,961,728 [0/0]	129,328,469 [0/0]	141,009,466 [0/0]
	Number of contigs	200	230	448	129
	Min (bp)	18,733	21,643	10,030	1,345
	Max (bp)	4,940,096	5,524,336	6,701,515	7,872,319,
	contig N50	1,907,605	1,866,906	1,356,898	3,954,136[NIFG]
	%GC	54	54	53	53
	Number of genes	13,860	14,050	12,903	13,455
	Gene density (genes/Mb)	104.2	104.9	99.7	95.4
	Repeats (%)*	26.82	26.82	25.85	33.88
	BUSCO scores**	C: 97.7% S: 95.9% D: 1.8% F: 0.3% M:2.0%	C: 94.9% S: 93.0% D: 1.9% F: 0.4% M:4.7%	C: 98.1% S: 96.4% D: 1.6% F: 0.5% M: 1.4%	C:98.1% S:97.2% D:0.9% F:1.0% M:0.9%
	Estimated genome size (Mbp)***	137.6	136.5	127.4	147.4

SDR or SDLR/ short SDLR	Size (Mbp)	1.01	0.98	1.02/0.20	0.98
	%GC	51	51	51/50	49
	Number of genes	28	28	30/4	34
	Gene density (genes/Mb)	27.64	28.82	29.44/20.00	34.7
	Repeats (%)*	70.15	70.20	74.83/84.83	59.04

*Repetitive sequences identified using RepeatMasker 4.1.4 (http://www.repeatmasker.org) with Dfam3.0, generated libraries of repeats by RepeatModeler2.0 (http://www.repeatmasker.org/RepeatModeler/ /).

**BUSCO v5.1.2[6] scores calculated based on chlorophyta_odb10 (1,519 BUSCO). C: complete; S: complete and single-copy; D: complete and duplicated; F: fragmented; M: missing.

***Based on *k-mer* profiles by GenomeScope.[1]

Table S3. Comparison of whole genome and SDR or SDLR/short SDLR properties of the volvocine algae*, Related to Figure 2 and Star Methods.

		Whole genome				
Species (strain)	Mating type/sex	Size/total length (Mb)	%GC	Number of genes	Gene density (genes/Mb)	DDBJ/ENA/ GenBank accessions no.
V. reticuliferus	Female	133	54	13860	104.2	BNCP01000001-BNCP01000200
	Male	134	54	14050	104.8	BNCQ01000001-BNCQ01000230
V. africanus (Japanese)	Homothallic	127	53	13716	108.1	BNCO01000001-BNCO01000448
V. africanus (Thai)	Homothallic	141	53	13455	95.4	BSDZ01000001-BSDZ01000129
V. carteri	Female	131.1	56.1	14958	114	GCA_000143455.1
	Male	N.d.	N.d.	N.d.**	N.d.	N.d.
Eudorina sp.	Female	184	61	N.d.	N.d.	GCA_003117195.1
	Male	168.6	61.3	N.d.	N.d.	GCA_003117095.1
Y. unicocca	Plus	134.2	61.1	N.d.	N.d.	GCA_003116995.1
	Minus	140.8	60.8	N.d.	N.d.	GCA_003117035.1
G. pectorale	Plus	149	64.5	17990	121	GCA_001584585.1
	Minus	N.d.	N.d.	N.d.	N.d.	N.d.
C. reinhardtii	Plus	111.1	64.1	17732	159.6	GCA_000002595.2
	Minus	N.d.	N.d.	N.d.	N.d.	N.d.

Table S3. Continued.

		SDR or SDLR/short SDLR				
Species (strain)	Mating type/sex	Size (Mb)	%GC	Number of genes	Gene density (genes/Mb)	DDBJ/ENA/GenBank accessions no.
V. reticuliferus	Female	1.01	51	28(25***)	27.7	LC586643
	Male	0.98	51	28(25)	28.6	LC586644

V. africanus (Japanese)	Homo-thallic	1.02	51/50	30/4	29.4/20	LC586641/ LC586642
V. africanus (Thai)	Homo-thallic	0.98	49	34	34.7	LC749599
V. carteri	Female	1.51	52	55(50)	39	GU784915.1
	Male	1.13	53	60(50)	54	GU784916.1
Eudorina sp.	Female	0.09	53.9	3(2)	33.3	LC314414.1
	Male	0.007	51.4	3(2)	428	LC314415.1
Y. unicocca	Plus	0.268	60.1	18(17)	67.2	LC314412.1
	Minus	0.165	60.3	18(17)	109	LC314413.1
G. pectorale	Plus	0.366	59.7	24(21)	58	LC062718
	Minus	0.499	61	24(21)	46	LC062719
C. reinhardtii	Plus	0.31	60	35(22)	109	GU814014.1
	Minus	0.204	61	25(22)	118	GU814015.1

*References: *V. africanus* from Thailand (the present study); *V. reticuliferus* and *V. africanus* from Japan (Yamamoto et al.[2]); *C. reinhardtii* (Ferris et al.[7]); *G. pectorale* (Hamaji et al.[8]); *Y. unicocca* and *Eudorina* sp. (Hamaji et al.[9]); *V. carteri* (Ferris et al.[7]).

**Not determined.

***The number of gametologs in parentheses.

Table S4. List of genes in SDR and pseudo autosomal regions of heterothallic *Volvox reticuliferus*[2] and their orthologs in Japanese and Thai culture strains of homothallic *V. africanus*, Related to Figures 1 and 2A. Genes in SDR or R domain in *V. reticuliferus* are shown in bold. Note that two genes (CGL55 and DHC1b, see below) and eight additional genes (*SPL2, ATS2*, *SelEF*, *MTM1037*, *DRG1, NMT1*, *VA_018_082* and *ATPvC1*) distributing in both SDLRs from Japanese and Thai strains (Figures 2A and S8) have homologs distributed in pseudo autosomal and autosomal regions of heterothallic *V. reticuliferus*, respectively.

V. reticuliferus gene	Ortholog in Japanese strain of *V. africanus*		Ortholog in Thai strain of *V. africanus*	
	Location	Name	Location	Name
THI10	autosome-like	THI10	autosome-like	THI10
VR_PAR_021	autosome-like	VA_PAR_021	autosome-like	VA_PAR_021
UTP1	autosome-like	UTP1	autosome-like	UTP1
HGRP1	autosome-like	HGRP1	autosome-like	HGRP1
PKY1	autosome-like	PKY1	autosome-like	PKY1
MADS2	autosome-like	mads2	autosome-like	mads2
UBCH1	autosome-like	UBCH1	autosome-like	UBCH1
GCSH	autosome-like	GCSH	autosome-like	GCSH
PR46b	autosome-like	PR46b	autosome-like	PR46b
RPL37A	autosome-like	RPL37A	autosome-like	RPL37A
BBS2	autosome-like	BBS2	autosome-like	BBS2
VR_PAR_033	-	-	-	-
VR_PAR_034	-	-	-	-
VR_PAR_035	-	-	-	-
ATPvL1	autosome-like	ATPvL1	autosome-like	ATPvL1
FTT2	autosome-like	FTT2	autosome-like	FTT2
UCP2	autosome-like	UCP2	autosome-like	UCP2
FA1	autosome-like	FA1	autosome-like	FA1
VR_PAR_041	autosome-like	VA_PAR_041	autosome-like	VA_PAR_041
VR_PAR_042	autosome-like	VA_PAR_042	autosome-like	VA_PAR_042
DNJ11	autosome-like	DNJ11	autosome-like	DNJ11*
CGL55	SDLR	CGL55	SDLR	CGL55
**VRMT001**	SDLR	VAMT001	SDLR	VAMT001
**VRMT003**	SDLR	VAMT003	SDLR	VAMT003
**MTM1058**	SDLR	MTM1058	SDLR	MTM1058

**MTM0349**	SDLR	MTM0349	SDLR	MTM0349
**MTM0417**	SDLR	MTM0417	SDLR	MTM0417
**MTD1****	autosome-like	MTD1	SDLR	MTD1
**VRM001****	-	-	SDLR	VRM001
**VRMT014**	SDLR	VAMT014	SDLR	VAMT014
**WDR57**	SDLR	WDR57	SDLR	WDR57
**VRMT030**	SDLR	VAMT030	SDLR	VAMT030
**TOC34**	autosome-like	TOC34	autosome-like	TOC34
**SPS1**	SDLR/short SDLR	SPS1/ψ-SPS1	SDLR	SPS1
**EIF5Bb**	SDLR	EIF5Bb	SDLR	EIF5Bb
**MAT3**	SDLR	MAT3	SDLR	MAT3
**CRB1**	SDLR	CRB1	SDLR	CRB1
**VRMT041**	autosome-like	VAMT041	autosome-like	VAMT041
**VRMT043**	SDLR	VAMT043	SDLR	VAMT043
**MTM0397**	SDLR/short SDLR	MTM0397/ψ-MTM0397	SDLR	MTM0397
**MME6**	autosome-like	MME6	SDLR	MME6
**MOT41**	autosome-like	MOT41	autosome-like	MOT41
**VRMT051**	SDLR/short SDLR	VAMT051/ψ-VAMT051	SDLR	VAMT051
**UNC50**	SDLR	UNC50	SDLR	UNC50
**MID****	short SDLR	MID	SDLR	MID
**MTM0637**	SDLR	MTM0637	SDLR	MTM0637
**MTM0638**	SDLR	MTM0638	SDLR	MTM0638
**PTC1**	SDLR	PTC1	SDLR	PTC1
**PSF2**	SDLR	PSF2	SDLR	PSF2
ARP4	autosome-like	ARP4	autosome-like	ARP4
L7Aef	autosome-like	L7Ae	autosome-like	L7Ae
MAPKK1	autosome-like	MAPKK1	autosome-like	MAPKK1
MTM0041	autosome-like	MTM0041	autosome-like	MTM0041
MTM0046	autosome-like	MTM0046	autosome-like	MTM0046
LEU1S	autosome-like	LEU1S	autosome-like	LEU1S
AMPKR1	autosome-like	AMPKR1	autosome-like	AMPKR1
EFG8	autosome-like	EFG8	autosome-like	EFG8

METM	autosome-like	METM	autosome-like	METM
HSP70b	autosome-like	HSP70b	autosome-like	HSP70b
BFR1	autosome-like	BFR1	autosome-like	BFR1
MTM0001	autosome-like	MTM0001	autosome-like	MTM0001
VR_PAR_087	autosome-like	VA_PAR_087	autosome-like	VA_PAR_087
PAP1	autosome-like	PAP1	autosome-like	PAP1
PIP5K1	-	-	-	-
FAP75	-	-	-	-
MT0045	autosome-like	Vafri_22190	autosome-like	VaTH14739
MT0044	autosome-like	Vafri_22189	autosome-like	VaTH14378
PRP4	autosome-like	PRP4	autosome-like	PRP4
DHC1b	SDLR	DHC1b	SDLR	DHC1b
**VRF001*** **	-	-	-	-
**VRF002*** **	-	-	-	-
**FUS1*** **	SDLR	ψ-FUS1	-	-

*Excluded from annotation (Figures 2, S2, S3 and S4) because the homologous sequence lacked apparent RNA expression.

**Male-specific gene.

***Female-specific gene.

Table S5. Models used for the Bayesian inference (BI) and maximum likelihood method (ML) in molecular phylogenetic analyses of homologs of fully sex-linked genes (male- and female-specific genes) and gametologs of *Volvox reticuliferus* and *V. africanus*, Related to Figures 2A and S5-S8, and Star Methods.

Gene name	BI_model*	ML_model*	Figure
ATPvC1	WAG+G	JTT+G	Figure S8
ATS2	WAG+G	WAG+G	Figure S8
CGL55	WAG	WAG+F	Figure S8
CRB1	Dayhoff+I+G	JTT+I+G+F	Figure S5
DHC1b	WAG+I+G	JTT+G	Figure S8
DRG1	WAG+G	JTT+G	Figure S8
EIF5Bb	Dayhoff+I+G	JTT+G+F	Figure S5
MAT3	Dayhoff+I+G	JTT+I+G+F	Figure S5
MID	WAG+G	JTT-DCMUT+G	Figure 2B
MME6	WAG+I+G	JTT+I+G	Figure S7
MOT41	Dayhoff+G	JTT+G	Figure S7
MTD1	WAG+I+G	JTT+G+F	Figure 2B
MTM0349	WAG+G	JTT+G	Figure S5
MTM0397	WAG+I+G	JTT-DCMUT+I+G	Figure S6
MTM0417	Dayhoff+G	JTT+G	Figure S5
MTM0637	Dayhoff+G	JTT+G	Figure S5
MTM0638	WAG+I+G	JTT+I+G+F	Figure S5
MTM1037	WAG+G	JTT+G+F	Figure S8
MTM1058	Dayhoff+G	JTT+G	Figure S5
NMT1	Dayhoff+I	JTT+I	Figure S8
PSF2	Dayhoff+I+G	JTT+I+G+F	Figure S5
PTC1	WAG+G	JTT+G	Figure 2B
SelEF	WAG+G	JTT+G	Figure S8
SPL2	VT+G	JTT+G	Figure S8
SPS1	WAG+G	JTT+G	Figure S6
TOC34	WAG+G	JTT+G	Figure S7
UNC50	MTREV+G	MTZOA+G	Figure S5
VAMT001	WAG+G	JTT+G+F	Figure S5
VAMT003	WAG+G	MTZOA+G+F	Figure S5
VAMT014	VT+G	HIVB+G+F	Figure S5

VAMT030	VT+G	LG+G	Figure S5
VAMT041	WAG+G	FLU+G+F	Figure S7
VAMT043	WAG+G	JTT+G	Figure S5
VAMT051	WAG+G	JTT+G	Figure S6
VA_018_082	VT+G	JTT+G	Figure S8
VRM001	WAG+G	JTT-DCMUT+G+F	Figure 2B
WDR57	WAG+G	JTT+G	Figure S5

*The best-fitted model was selected by ModelTest-NG 0.1.6.[10]

SI References

1. Vurture, G. W., Sedlazeck, F. J., Nattestad, M., Underwood, C. J., Fang, H., Gurtowski, J., and Schatz, M. C. (2017). GenomeScope: fast reference-free genome profiling from short reads. Bioinformatics, 33, 2202–2204. https://doi.org/10.1093/bioinformatics/btx153.
2. Yamamoto, K., Hamaji, T., Kawai-Toyooka, H., Matsuzaki, R., Takahashi, F., Nishimura, Y., Kawachi, M., Noguchi, H., Minakuchi, Y., Umen, J. G., Toyoda, A., and Nozaki, H. (2021). Three genomes in the algal genus *Volvox* reveal the fate of a haploid sex-determining region after a transition to homothallism. Proc. Natl. Acad. Sci. U S A 118, e2100712118. https://doi.org/10.1073/pnas.2100712118.
3. Starr, R. C. (1971). Sexual reproduction in *Volvox africanus*. In: Contribution in Phycology, B. C. Parker and R. M. Brown Jr., eds. (Allen Press, Kansas), pp 59–66.
4. Nozaki, H., Matsuzaki, R., Yamamoto, K., Kawachi, M., and Takahashi, F. (2015). Delineating a new heterothallic species of *Volvox* (Volvocaceae, Chlorophyceae) using new strains of "*Volvox africanus*". PloS one, 10, e0142632. https://doi.org/10.1371/journal.pone.0142632.
5. Nozaki, H., Mahakham, W., Heman, W., Matsuzaki, R., and Kawachi, M. (2022). Morphology, mating system and taxonomy of *Volvox africanus* (Volvocaceae, Chlorophyceae) from Thailand. Bot. Stud. 63, 1. https://doi.org/10.1186/s40529-022-00332-1.
6. Manni, M., Berkeley, M. R., Seppey, M., Simão, F. A., and Zdobnov, E. M. (2021). BUSCO update: Novel and streamlined workflows along with broader and deeper phylogenetic coverage for scoring of eukaryotic, prokaryotic, and viral genomes. Mol. Biol. Evol. 38, 4647–4654. https://doi.org/10.1093/molbev/msab199.
7. Ferris, P., Olson, B. J., De Hoff, P. L., Douglass, S., Casero, D., Prochnik, S., Geng, S., Rai, R., Grimwood, J., Schmutz, J., Nishii, I., Hamaji, T., Nozaki, H., Pellegrini, M., and Umen, J. G. (2010). Evolution of an expanded sex-determining locus in *Volvox*. Science, 328, 351–354. https://doi.org/10.1126/science.1186222.
8. Hamaji, T., Mogi, Y., Ferris, P. J., Mori, T., Miyagishima, S., Kabeya, Y., Nishimura, Y., Toyoda, A., Noguchi, H., Fujiyama, A., Olson, B. J., Marriage, T. N., Nishii, I., Umen, J. G., and Nozaki, H. (2016). Sequence of the *Gonium pectorale* mating locus reveals a complex and dynamic history of changes in volvocine algal mating haplotypes. G3 (Bethesda) 6, 1179–1189. https://doi.org/10.1534/g3.115.026229.
9. Hamaji, T., Kawai-Toyooka, H., Uchimura, H., Suzuki, M., Noguchi, H., Minakuchi, Y., Toyoda, A., Fujiyama, A., Miyagishima, S. Y., Umen, J. G., and Nozaki, H. (2018). Anisogamy evolved with a reduced sex-determining region in volvocine green algae. Commun. Biol. 1, 17. https://doi.org/10.1038/s42003-018-0019-5.

10. Darriba, D., Posada, D., Kozlov, A. M., Stamatakis, A., Morel, B., and Flouri, T. (2020). ModelTest-NG: A new and scalable tool for the selection of DNA and protein evolutionary models. Mol. Biol. Evol. 37, 291–294. https://doi.org/10.1093/molbev/msz189.
